# Metagenomic analysis for the microbial consortium of anaerobic CO oxidizers

**DOI:** 10.1111/1751-7915.12283

**Published:** 2015-04-15

**Authors:** Ying Guo, Jingliang Xu, Zhenhong Yuan, Xiekun Li, Weizheng Zhou, Huijuan Xu, Cuiyi Liang, Yu Zhang, Xinshu Zhuang

**Affiliations:** 1 Key Laboratory of Renewable Energy, Guangzhou Institute of Energy Conversion, Chinese Academy of SciencesGuangzhou, 510640, China; 2 Graduate University of Chinese Academy of SciencesBeijing, 100049, China

## Abstract

Metagenomics analysis has been applied to identify the dominant anaerobic microbial consortium of the carbon monoxide (CO) oxidizers in anaerobic sludge. Reads from the hypervariable V6 region in the bacterial 16s rDNA were aligned and finally clustered into operational taxonomic units (OTUs). The OTUs from different stages in anaerobic CO condition were classified. Alphaproteobacteria, clostridia, betaproteobacteria and actinobacteria were the most abundant groups, while alphaproteobacteria, betaproteobacteria and actinobacteria were variable groups. CO consumption and production efficiency of the microbial consortium were studied. Semi-continuous trials showed that these anaerobic CO oxidizers formed a stable microbial community, and the CO conversion rate was at over 84%, with the highest CO consumption activity of 28.9 mmol CO/g VSS●day and methane production activity at 7.6 mmol CH_4_/g VSS●day during six cycles.

## Introduction

Recent measurements indicate that the natural atmospheric carbon monoxide (CO) concentrations have risen greatly over the course of industrial development. Incomplete combustion of carbon-based fuels in industrial processes create over 3.3 × 10^9^ metric ton CO per year, besides originating routinely from numerous biological production and degradation (Haab, 1990; Conrad, 1996). The presence of CO connects the microbiology community instead of merely algae and plants.

CO microbial oxidizer is essential to the global carbon cycle. Improvements in metagenomic analyses technology provide a robust tool to determine the microbial community response to accelerated CO levels. 16S rDNA analogous based on specific primers has been successfully applied to determine aerobic CO oxidizers *in situ* with primers for the carbon monoxide dehydrogenases (CODHs) (Dunfield and King, 2004; Weber and King, 2010; Yang *et al*., 2013). The anaerobic microbial groups using elevated CO concentrations have not been addressed yet. Due to lack of this part of study, the diversity and phylogenetic affiliations of CO oxidizer remain unclear (King and Weber, 2007).

The insight into the anaerobic CO oxidizers population structure as well as their metabolic interactions should be beneficial for analyses of diversity and the manipulation of microbiology. In the anaerobic microbiology community, CO is reported to work as carbon and energy source through the Wood–Ljungdahl pathway and CODH, and thus becomes a valuable substrate for biomass and fuel (Ragsdale, 2007; Kim and Park, 2012). CODH contributes to the reversible conversion between CO and carbon dioxide (CO_2_). These anaerobic Ni-CODHs are 1000-fold higher than the Mo-CODHs in the aerobic microbes (Ragsdale, 2004). CO and CO_2_ enter into the Wood–Ljungdahl pathway from carbonyl and methyl branch, respectively, to form acetyl-CoA with acetyl-CoA synthase (ACS). Acetyl-CoA can be used as growth or energy varied between the species (Martin and Russell, 2007; Seravalli and Ragsdale, 2008; Ragsdale *et al*., 2012).

Our study has been conducted to demonstrate the metagenomic analysis on the high-efficiency anaerobic CO microbial groups and mediate feedbacks to exceed CO levels. The experimental modelling of anaerobic conditions has been applied to study the abundance and diversity of microbial CO oxidizers. In the present study, we investigate the responses of the microbial community to the artificial anaerobic CO atmosphere using 16S rDNA gene sequence analysis first, and the stability of those active groups was conducted then.

## Results and discussion

### The potential of CO conversion

The active anaerobic CO oxidizers in microbial community were studied. For the sewage sludge, first a reviving stage for CO consumption in the initial 5 days was run, with the headspace gases comprising a small amount of CH_4_, CO_2_ and trace H_2_ (less than 0.5%). The batch culture was then operated at a stable CO consumption rate for the next 12 days. During the second stage, the gas bag system showed comparable performance and process efficiency. CO was fully converted into CH_4_ and CO_2_. In the third stage, the carbon source totally relied on CO_2_. CO_2_ was forced into CH_4_ or acids, and a total of 63%–70% of the CH_4_ were obtained in the headspace gas, with an eventual build-up 7.1–6.5 mmol L^−1^ acetate and 4.9–2.8 mmol L^−1^ butyrate by day 63. We estimated that the performance of microbial community was divided into three stages: the reviving, efficient and stable stages.

### 16S rDNA gene sequence analysis

#### Samples fetched in different stages were conducted with 16S rDNA gene sequence analysis

Reads from the hypervariable V6 region in the bacterial 16s rDNA were clustered into operational taxonomic units (OTUs). Overall, a total of 3350, 11721, 2968 and 1957 unique tags were generated from the stable, efficient and reviving stages. and original cultured sludge, with 908, 4677, 1072 and 539 OTUs respectively.

**Figure 1:**
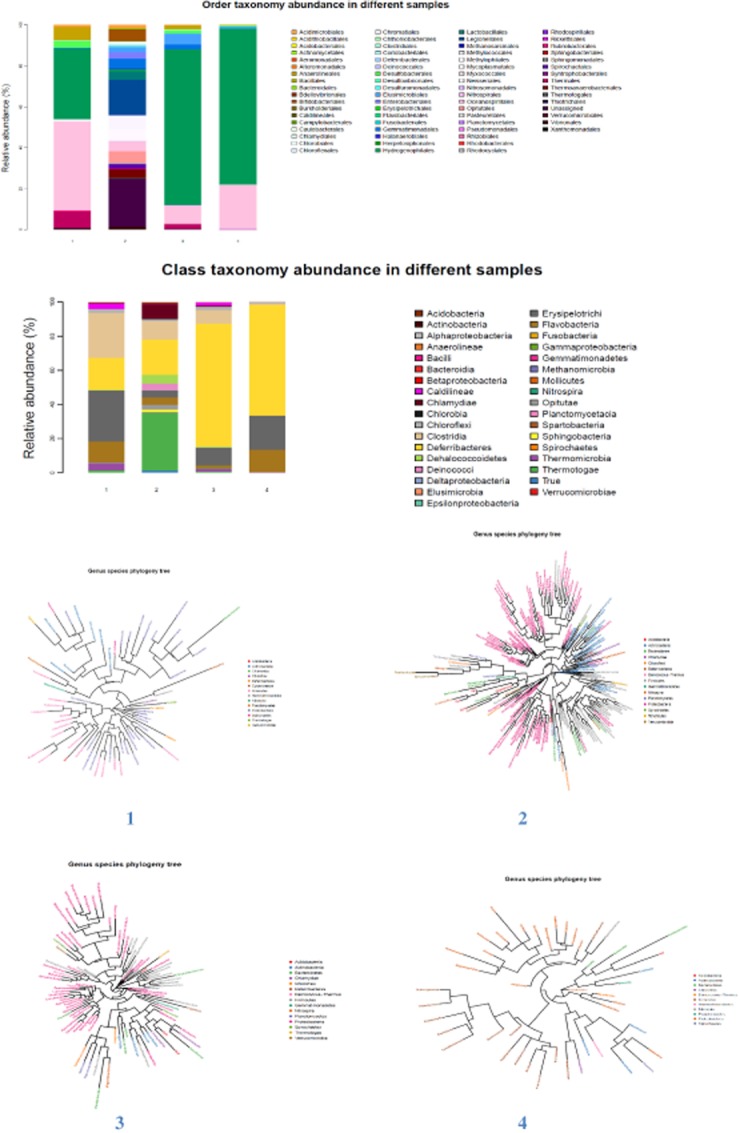
Taxonomy and phylogenetic trees of different stages: 1 (stable stage), 2 (efficient stage), 3 (reviving stage), 4 (cultured sludge).

The dominant represented anaerobic CO oxidizers were determined to be the active groups in betaproteobacteria and actinobacteria. Though less abundance, the clostridia and alphaproteobacteria still play a major role in the anaerobic CO consumption pathways. These estimated phylogenetic trees and taxonomy compositions are illustrated in Fig. 1 and Table 1.

**Table 1:** Number of OTUs per bacterial class

Class	1 (the stable stage)		2 (the efficient stage)		3 (the reviving stage)		4 (cultured sludge)	
	OTUs	%	OTUs	%	OTUs	%	OTUs	%
Methanomicrobia	1	0.00	0	0	0	0	0	0
Acidobacteria	142	0.12	1305	1.11	93	0.08	15	0.01
Actinobacteria	767	0.64	32658	27.73	244	0.21	68	0.06
Bacteroidia	2728	2.29	234	0.20	1265	1.07	191	0.17
Flavobacteria	1	0.00	92	0.08	4	0.00	1	0.00
Sphingobacteria	2	0.00	1343	1.14	18	0.02	1	0.00
Chlamydiae	11	0.01	281	0.24	61	0.05	1	0.00
Chlorobia	0	0	0	0	2	0.00	1	0.00
Anaerolineae	215	0.18	2559	2.17	208	0.18	12	0.01
Caldilineae	0	0	1	0.00	0	0	0	0
Chloroflexi	1	0.00	12	0.01	0	0	0	0
Dehalococcoidetes	1	0.00	2	0.00	1	0.00	0	0
TRUE	44	0.04	19	0.02	4	0.00	0	0
Deferribacteres	51	0.04	94	0.08	126	0.11	0	0
Deinococci	0	0	12	0.01	7	0.01	50	0.04
Bacilli	8097	6.80	4204	3.57	899	0.76	11085	9.59
Clostridia	19321	16.22	3704	3.14	7233	6.12	16998	14.71
Erysipelotrichi	0	0	213	0.18	33	0.03	25	0.02
Fusobacteria	0	0	1	0.00	0	0	0	0
Gemmatimonadetes	4	0.00	73	0.06	32	0.03	1	0.00
Nitrospira	10	0.01	3829	3.25	13	0.01	2	0.00
Planctomycetacia	400	0.34	4827	4.10	51	0.04	2	0.00
Alphaproteobacteria	12142	10.19	19950	16.94	49494	41.89	55196	47.77
Betaproteobacteria	17307	14.53	10543	8.95	5410	4.58	751	0.65
Deltaproteobacteria	1356	1.14	1158	0.98	1390	1.18	493	0.43
Epsilonproteobacteria	2	0.00	85	0.07	13	0.01	0	0
Gammaproteobacteria	76	0.06	8317	7.06	539	0.46	50	0.04
Spirochaetes	2202	1.85	25	0.02	1207	1.02	91	0.08
Elusimicrobia	0	0	46	0.04	0	0	2	0.00
Mollicutes	0	0	11	0.01	0	0	0	0
Thermomicrobia	0	0	51	0.04	0	0	0	0
Thermotogae	2	0.00	0	0	1	0.00	0	0
Opitutae	3	0.00	5	0.00	16	0.01	0	0
Spartobacteria	0	0	49	0.04	0	0	0	0
Verrucomicrobiae	327	0.27	838	0.71	34	0.03	17	0.01

In the present study, CO_2_, CH_4_, acetate and butyrate were the main end-product, together with less than 0.5% H_2_.

The major aerobic CO oxidizers determined in situ were proteobacteria, actinobacteria and firmicutes (Dunfield and King, 2004; Weber and King, 2010; Yang *et al*., 2013). Our results were consistent with these observations as we found that alphaproteobacteria (proteobacteria), clostridia (firmicutes), betaproteobacteria (proteobacteria) and actinobacteria were the dominant groups in anaerobic conditions. These classes containing species with putative CODH (cox) genes could functionally cooperate in anaerobic conditions. The Wood–Ljungdahl pathway would be predicted to associate the anaerobic active groups with acetyl-CoA. Based on individual reported species, the acetogenic bacteria could participate in converting acetyl-CoA to acetates, with phosphate acetyl transferase and acetate kinase (Schiel-Bengelsdorf and Duerre, 2012), and in some cases two fatty acid chains were then added after the backbone acetate to form butyrate (Jang *et al*., 2012). Members of methanogenic species, such as *Methylobacterium* spp., were predicted to be involved in methane production. Methyl groups from the corrinoid iron–sulfur protein component of acetyl-CoA decarbonylase synthase or the methanogenic CODH/ACS complex could be turned to methyl-[Co], then methyl-SCoM and finally CH_4_. MeTr, heterodisulfide reductase and methyl-SCoM reductase, in methanogenic species, catalyzed these reactions. Two electrons were reported to be channelled to the heterodisulfide reductase for reducing the heterodisulfide product (CoB-SS-CoM) to methyl-SCoM reductase (HSCoB) (Shima *et al*., 2002; Ragsdale and Pierce, 2008).

**Figure 2:**
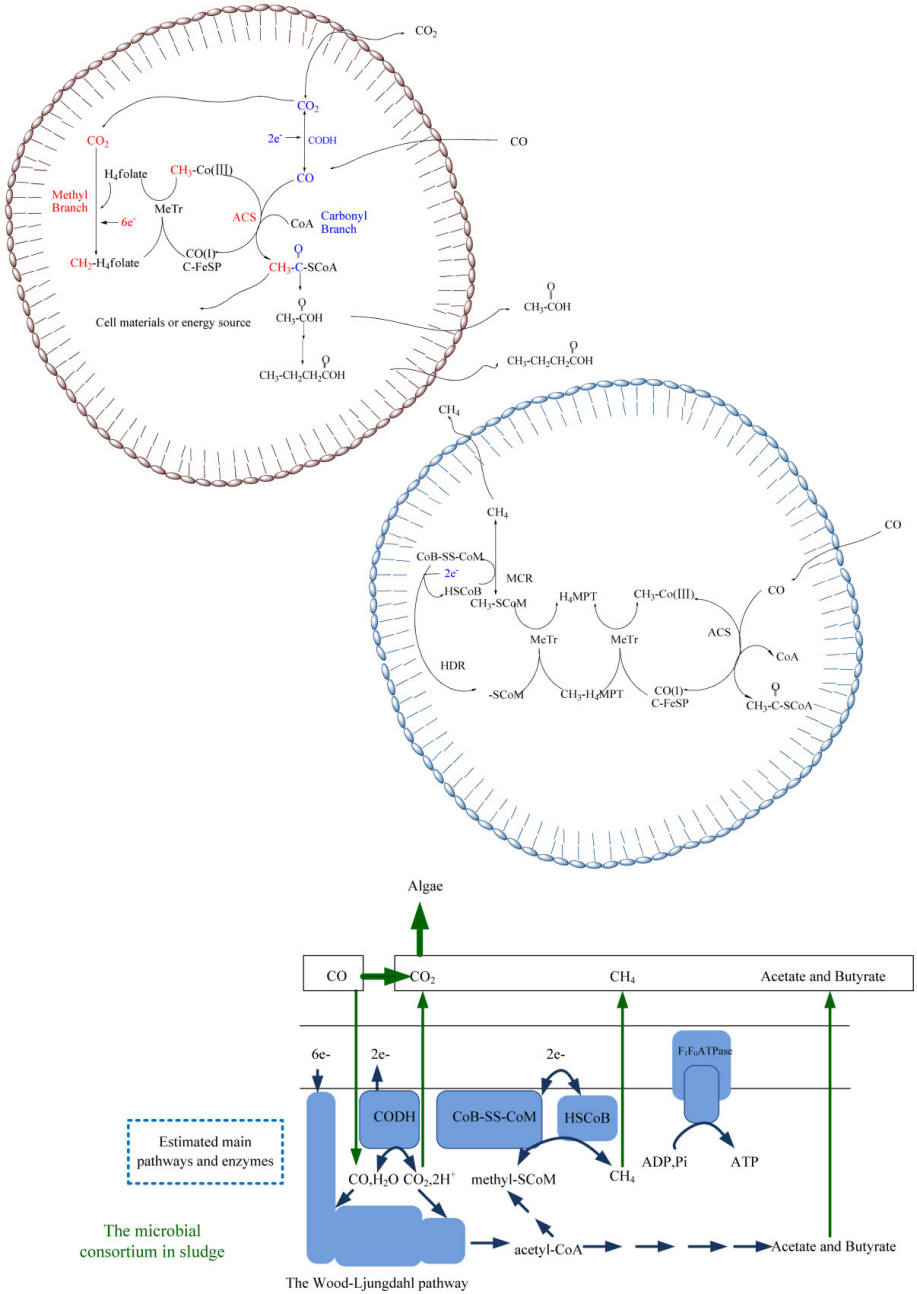
Predicted pathways inside microbial systems for conversion of carbon monoxide to carbon dioxide, methane and acids.

The reaction for converting CO substrates to CO_2_ by CODHs might be the main rate-limiting step. Electrons produced in this step could then be used for the methyl branch of the Wood–Ljungdahl pathway and reduce CoB-SS-CoM to HSCoB. Thus, when CO_2_ becomes the only substrate, few active electrons are available and extra electron donors should be added to enhance efficiency in the final stable stage. It was reported that CO_2_ could be utilized by microalgae, macroalgae or cyanobacteria instead of a CO oxidizer (Farrelly *et al*., 2013), before or after selective separation of CH_4_. The estimated main pathways and enzymes inside microbial systems for conversion of CO to CO_2_, CH_4_ and acids are illustrated in Fig. 2.

Treatment for CO with active bacterial groups should therefore be possible, though the accurate enzyme activity inside pathways still remains to be determined. These results highlighted some of the difficulties in predicting gas consumption and production efficiency with plenty of CO and electrons available in the long term. This also confirmed the need to carry out semi-continuous simulation trials to obtain an accurate CO consumption rate and thus to further verify whether the method developed in the study establishes a stable microbial system.

### Semi-continuous gas bag trial

**Figure 3:**
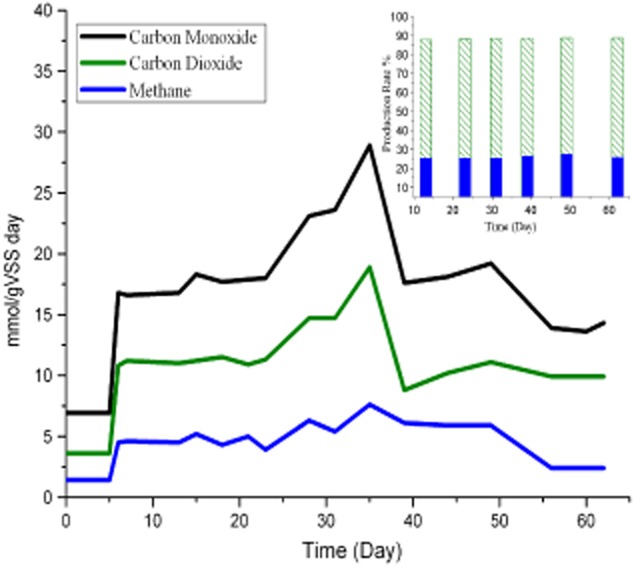
The speeds of carbon monoxide consumption, carbon dioxide and methane production, and the production rate of carbon monoxide for methane and carbon dioxide. mmol/gVSS day.

Experimental results for the semi-continuous trials are shown graphically in Fig. 3. The semi-continuous study reconfirmed that CO, an inhibitor to most organisms, could serve as a suitable feedstock for the microbial. Six experimental cycles were performed, and the speeds for CO consumption, CO_2_ and CH_4_ productions and CO conversion efficiency are illustrated in Figs 2 and 3. After 5 reviving days, gas bag systems ran at a stable CO consumption rate for another 8 days. Again, the third, fourth, fifth and sixth cycles were performed gradually. During these cycles, the gas bag system for the semi-continuous run was stable, with the highest CO consumption activity of 28.9 mmol CO/g VSS●day [mmol CO consumed by 1 g volatile suspended solids (VSS) per day], and CH_4_ production activity at 7.6 mmol CH_4_/g VSS●day. The CO conversion rate retained its activity and stayed over 84%, at all the phases, though, with a slightly lower efficiency of 71% in the first 5 reviving days. The highest CO consumption and CH_4_ production rate occurred in the fourth cycle from days 31 to 39. There was a build-up of acids reaching 6.4 mmol L^−1^ acetate and 3.5 mmol L^−1^ butyrate at the end of the experiment.

CODH and CH_4_-producing enzymes were predominant in the semi-continual trials. For CO_2_ and CH_4_, these almost accounted for 53% and 30% of the end-products, respectively, with just 4% acids and less than 0.5% H_2_. During six cycles, these anaerobic consortium with activated enzymes and pathways showed their stability and effectiveness. The highest CO consumption and CH_4_ production rate occurred at the phase when electrons were at the highest level.

### Conclusion

The 16S rDNA gene sequence analysis has been applied to identify the profile of anaerobic CO oxidizers in microbiology community. To verify the ability of functionally cooperated CO converting microorganisms, the potential of the microbial consortium in anaerobic sludge was studied. The cooperative microbial community with flexible pathways was a stable system after six cycles. This study confirmed that alphaproteobacteria (proteobacteria), clostridia (firmicutes), betaproteobacteria (proteobacteria) and actinobacteria were the dominant CO oxidizers. These classes could functionally cooperate and establish a stable and efficient microbial system in anaerobic conditions.

## Experimental procedures

### Inoculum and medium

The activated sludge (suspending solids, 4.7 g/L) was obtained from Datansha Wastewater Treatment Plant (of anaerobic process for treating municipal wastewater in Guangzhou, China). As soon as the samples were fetched from the factory, they were precipitated and revived in modified medium 640 and the final concentration of the volatile suspended solids (VSS) per litre medium (g VSS/L) was 1.9. Two hundred millilitre of medium was then injected into 2000 ml gas bags which were filled with CO at a gauge pressure of 1.0 atm. Before sterilization (20 min, 121°C), medium 640 was placed in an anaerobic chamber for 24–36 h to keep anaerobic. A detailed characteristics of modified medium 640 excluding carbon source were reported in our former study (Guo *et al*., 2010).

### Gas bag systems

**Figure 4:**
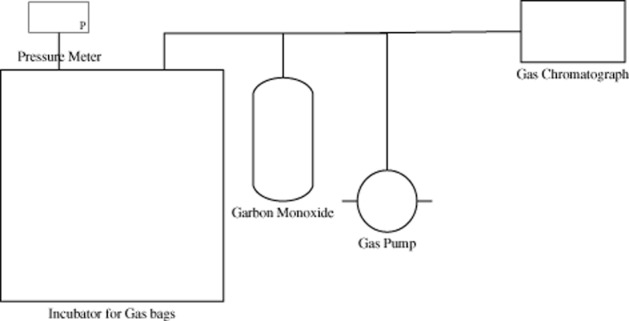
Typical schematic of a gas bag system.

The gas bag system was devised to perform the CO consumption of anaerobic microbial consortium. The advantages of this system are easy refill of gases free from contamination and a stable pressure even with the consumption of gases. Thus, gas bags do not need strict requirements for pressure or stirring. During the research, gas bags with a capacity of 2000 ml in the system are made of polyvinyl fluoride; could be connected with a pressure meter, cylinder, gas pump and GC; and were incubated at a temperature of 37°C. The volumes of the gas bags were obtained by recording the water volumes when inserting bags into water bath at 37°C. The typical schematic of the gas bag system is illustrated in Fig. 4.

### 16S rDNA gene sequence analysis

Total genomic DNA was extracted from samples using the Power Water™ DNA isolation kit following the manufacturer’s instructions. A portion of the bacterial 16S rDNA gene with hypervariable V6 region was then used as a template to amplify by polymerase chain reaction (PCR) with a standard thermal cycler. Those PCR products were purified with a purification kit and the sequencing was performed with the Illumina Hiseq2000 platform after verification by the agarose gel electrophoresis. Non-chimeric sequence reads from the Illumina Hiseq2000 platform were analysed and finally clustered into OTUs with the software Mothur (version 1.27.0). The taxonomic analysis of OTUs were performed with the software BLAST (version 2.2.23) using the SILVA database to determine the phylogenetic affiliations of microbial community (http://www.vamps.mbl.edu/resources/databases.php).

### Gas bag performance

The potential gas consumption and production efficiency of the microbial consortium were studied. To verify the stability of microbial systems, studies were conducted for six cycles. In all cases, the headspace gases and end-products of medium were analysed.

### Analytical methods

Gas composition was measured by gas chromatograph Agilent 7890 GC (Agilent Technologies, USA) with a thermal conductivity detector (TCD) and a 2 m stainless column packed with Porapak Q (50/80 mesh). Liquid samples were centrifuged with 10000 *g* for 10 min at 4°C for determination of C_2_-C_5_ acetate and alcohol concentrations of the supernatant. They were analysed using Agilent 6820 GC equipped with a flame ionization detector (FID) and a 30 m × 0.25 mm × 0.25 μm capillary column (DB-FFAP).

## Conflict of Interest

None declared.
